# Regulation of Flagellar Biogenesis by a Calcium Dependent Protein Kinase in *Chlamydomonas reinhardtii*

**DOI:** 10.1371/journal.pone.0069902

**Published:** 2013-07-25

**Authors:** Yinwen Liang, Junmin Pan

**Affiliations:** Ministry of Environment Key Laboratory of Protein Science, School of Life Sciences, Tsinghua University, Beijing, China; Institute of Molecular and Cell Biology, Singapore

## Abstract

*Chlamydomonas reinhardtii*, a bi-flagellated green alga, is a model organism for studies of flagella or cilia related activities including cilia-based signaling, flagellar motility and flagellar biogenesis. Calcium has been shown to be a key regulator of these cellular processes whereas the signaling pathways linking calcium to these cellular functions are less understood. Calcium-dependent protein kinases (CDPKs), which are present in plants but not in animals, are also present in ciliated microorganisms which led us to examine their possible functions and mechanisms in flagellar related activities. By in silico analysis of *Chlamydomonas* genome we have identified 14 CDPKs and studied one of the flagellar localized CDPKs – CrCDPK3. CrCDPK3 was a protein of 485 amino acids and predicted to have a protein kinase domain at the N-terminus and four EF-hand motifs at the C-terminus. In flagella, CrCDPK3 was exclusively localized in the membrane matrix fraction and formed an unknown 20 S protein complex. Knockdown of *CrCDPK3* expression by using artificial microRNA did not affect flagellar motility as well as flagellar adhesion and mating. Though flagellar shortening induced by treatment with sucrose or sodium pyrophosphate was not affected in RNAi strains, CrCDPK3 increased in the flagella, and pre-formed protein complex was disrupted. During flagellar regeneration, CrCDPK3 also increased in the flagella. When extracellular calcium was lowered to certain range by the addition of EGTA after deflagellation, flagellar regeneration was severely affected in RNAi cells compared with wild type cells. In addition, during flagellar elongation induced by LiCl, RNAi cells exhibited early onset of bulbed flagella. This work expands new functions of CDPKs in flagellar activities by showing involvement of CrCDPK3 in flagellar biogenesis in *Chlamydomonas* .

## Introduction

*Chlamydomonas reinhardtii*, a green alga, has been used as a model system for studies of various cellular processes [1]. Unlike higher plants, *Chlamydomonas* possesses two flagella, which are essentially identical to cilia present in animal kingdoms [2]. In vertebrates, primary cilia are generally immotile though with a few exceptions (e.g. nodal primary cilia are motile) and function by transmitting and processing mechanical, chemical and developmental cues [3], [4] [5], [6],. Motile cilia are involved in cell motility to propel cell motion such as sperm swimming or drive fluid flow in the brain and trachea [7]. In *Chlamydomonas* , flagella are employed for both cell motility and signaling in mating [1].

Calcium, a universal second messenger, has been reported to be intimately involved in a variety of flagellar related activities including phototaxis as well as flagellar beating [8], [9], flagellar gliding [10], deflagellation [11] and mating which depends on flagellar adhesion [12], [13], flagellar outgrowth and shortening [14], [15] [16],

One mechanism by which calcium exerts its divergent regulation of flagellar activities is through phosphorylation of flagellar proteins. Phosphoproteomic analysis of flagellar proteins has identified protein kinases and phosphatases [17], [18]. In vitro assay of protein phosphorylation of flagellar proteins has identified a set of proteins whose phosphorylation is regulated by calcium [19]. In animals, calcium-dependent protein phosphorylation is mediated by calcium/calmodulin-dependent kinases (CaMK) [20] and PKC [21]. Interestingly, CaMK are rare [22] and PKCs are not found in plants [23]. Instead, plants have a large family of calcium-dependent kinases (CDPKs), which harbor both protein kinase domain and calmodulin-like domain in one single molecule [24], [25]. Interestingly, a large number of CDPKs are also present in ciliated microorganisms including *Plasmodium, Tetrahymena,* *Paramecium* and *Chlamydomonas* [26]. Flagellar or ciliary localization of CDPKs has been reported in *Paramecium* [27], [28], and green algae *C* *. eugametos* [29] and *C. reinhardtii* [30] while their physiological functions remain unknown.

Using in silico analysis, we have identified 14 CDPKs in *C. reinhardtii*. CDPK1, 3 and 11 have been identified in *Chlamydomonas* flagellar proteome [30]. Here, we have studied physiological functions of CrCDPK3 in flagellar related activities and provided evidence that CrCDPK3 is involved in flagellar biogenesis.

## Results

### CDPKs in *C. reinhardtii*

**Figure 1:**
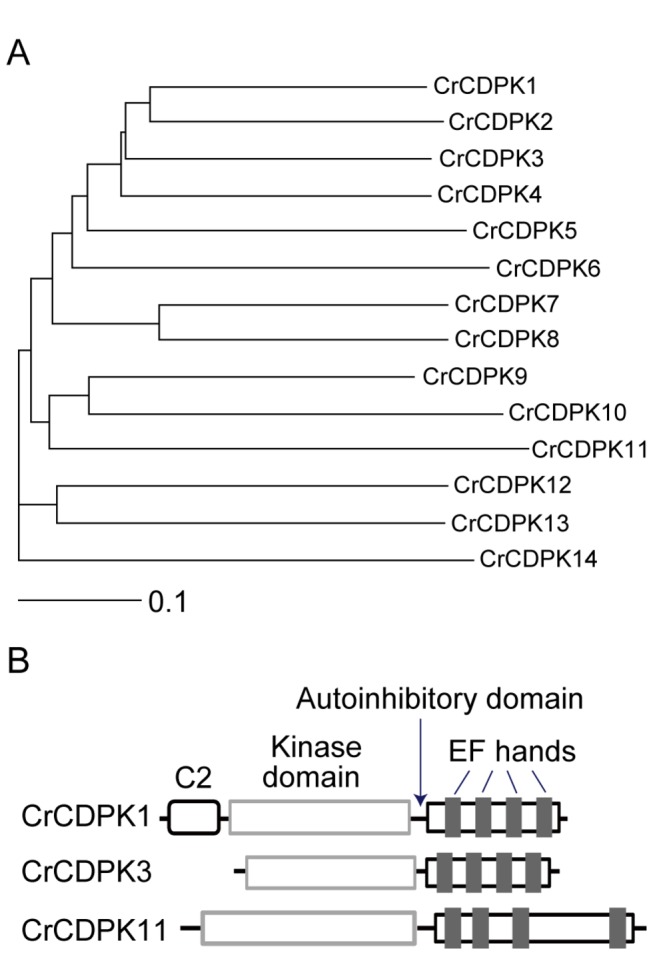
*C. reinhardtii* CDPKs. (A) Relatedness of CDPKs in *Chlamydomonas* . Protein sequences of the identified CDPKs annotated in *C. reinhardtii* genome v4 were aligned by using clastalx-2.1 and analyzed by the Phylip program (http://evolution.genetics.washington.edu/phylip.html). The branch lengths are proportional to divergence with the scale of “0.1” representing 10% change. (B) Schematic diagram of protein domains of three CrCDPKs identified in the flagellar proteome.

CDPKs are unique among calcium sensors because they combine calcium sensing and decoding within one single molecule with a kinase domain at the N-terminus and several calcium-binding EF-hand domains at the C-terminus. To identify CDPKs in *C. reinhardtii*, the cloned CDPK in *C* *. moewusii* [29] was used as query to search *Chlamydomonas* genome. 14 CDPKs were identified that had unique CDPK features. As summarized in Table 1, these CDPKs have various numbers of EF-hand motifs. A phylogenetic tree was built for the CDPKs identified (Figure 1A). Since a systematic naming for these kinases has not been made in the *Chlamydomonas* genome, we took liberty of naming these kinases according to relatedness in phylogenetic analysis. Thus, the naming order of these kinases does not necessarily indicate any physiological relevance. Previous microarray analysis of gene expression during flagellar regeneration has identified several CDPKs that show various extent of induction (Table 1). Three CDPKs including CDPK1, 3 and 11 are present in the flagellar proteome [30]. All three have four EF-hand motifs at the C-terminus, similar to canonical CDPKs in plants (Figure 1B) [31].

**Table 1:** CrCDPKs in *C. reinhardtii*.

	ID in Cr. V4	Name in Cr. V2	Amino acids	Number of EF hands	Expression (30min)	Flagellar Proteome
CrCDPK1	128451	C_170065	614	4	-23%	+
CrCDPK2	403209	ND	499	4	ND
CrCDPK3	127871	C_450030	484	4	+9.9%	+
CrCDPK4	377204	C_20322	526	4	-0.6%
CrCDPK5	406326	C_760001	632	4	+56%
CrCDPK6	525795	ND	50.79	3	ND
CrCDPK7	39553	ND	520	3	ND
CrCDPK8	416369	C_660009	469	3	-2%
CrCDPK9	316236	C_210091	425	4	-20%
CrCDPK10	149911	C_670016	590	2	-11%
CrCDPK11	413836	C_510021	1005	4	+35%	+
CrCDPK12	294983	C_270083	636	3	-4%
CrCDPK13	345104	C_70168	687	2	+7%
CrCDPK14	346299	C_380065	871	2	-37%

The protein ID or gene model name of each CDPK in *Chlamydomonas* genome v4 and v2 are shown, respectively. The numbers of EF-hand motifs were predicted with the SMART algorithm (http://smart.embl-heidelberg.de/). Data for induction of gene expression during flagellar regeneration [75] and presence in the flagellar proteome are included [30].

### CrCDPK3 is a flagellar membrane/matrix protein

**Figure 2:**
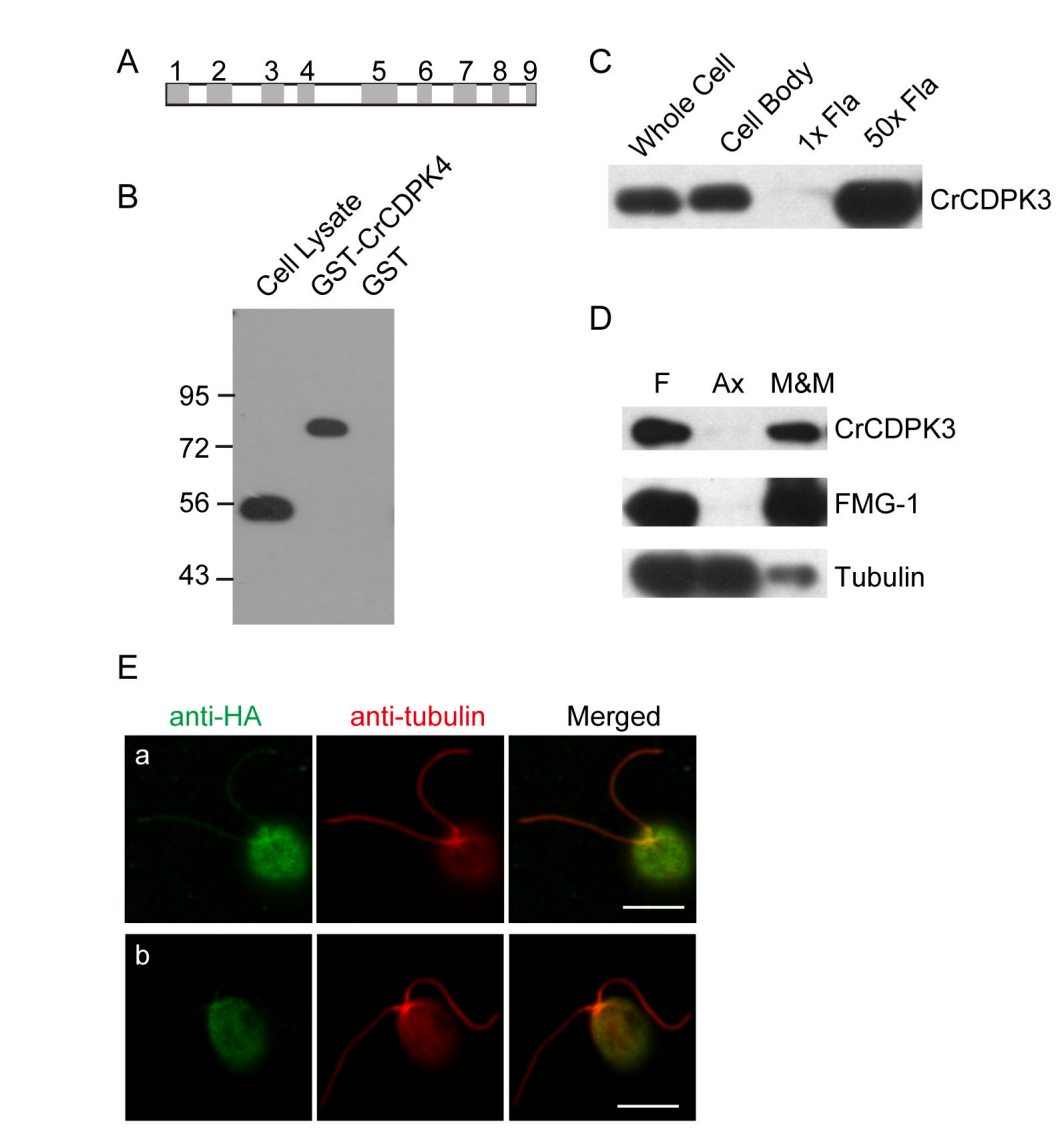
CrCDPK3 is present in the flagella of *C. reinhardtii*. (A) Schematic diagram of CrCDPK3 gene showing exons (grey) and introns (white). (B) Immunoblot analysis of *Chlamydomonas* cell lysates, bacterial expressed GST-CrCDPK3 and GST shows that anti-CrCDPK3 antibody is specific. Molecular weights are given in kilo-daltons. (C) CrCDPK3 is present in the cell body and flagella evidenced by immuoblotting with anti-CrCDPK3 antibody. 1 x indicates that approximately two flagella were loaded per cell body. 50 x indicates equal flagellar and cell body protein. (D) Isolated flagella (F), membrane/matrix (M and M) and axonemal (Ax) fractions were analyzed by immunoblotting with antibodies as indicated. (E) Immunostaining of cells expressing CrCDPK3-HA (a) or not (b) with antibodies against 3xHA tag and α-tubulin. Bars, 5 µm.

CDPKs present in the flagellar proteome are likely to function in flagellar related activities. CrCDPK3 was chosen for further studies. *CrCDPK3* is a gene of 3603 nucleotides with 9 exons and encodes a protein of 484 amino acids (Figure 2A). This annotation was confirmed after cDNA cloning and sequencing (see methods). SMART algorithm (http://smart.embl-heidelberg.de/) predicted a protein kinase domain at amino acid position 27-285, and four EF-hand motifs at positions 332-360, 368-396, 404-432 and 437-465, respectively (Figure 1B). To further study CrCDPK3, a polyclonal antibody was raised against the N-terminal 202 amino acids of CrCDPK3. Immunoblot analysis showed that this antibody was specific (Figure 2B). It recognized GST-tagged CrCDPK3 but not GST, and detected a single band in *Chlamydomonas* cell lysate with molecular weight of around 55 kD, similar to the predicted molecular weight of 53.98 kD.

To confirm flagellar presence of CrCDPK3, *Chlamydomonas* flagella were isolated and subjected to immunoblot analysis together with whole cell and cell body. CrCDPK3 was detected both in the cell body and flagella (Figure 2C). Flagellar proteins are approximately 2% of total cellular proteins [32]. When equal amounts of protein from cell body and flagella (50 x flag.) were loaded, it showed enrichment of CrCDPK3 in the flagella. We next examined the distribution of CrCDPK3 in the flagellar fractions. Isolated flagella were fractionated into membrane/matrix and axonemal fractions followed by SDS PAGE and immunoblot analysis. As expected, FMG1, a flagellar membrane protein [33], was solely localized in the membrane/matrix fraction, and tubulin was predominantly in the axonemal fraction. In contrast, CrCDPK3 was present only in the membrane/matrix. Since no transmembrane domain as well as lipid modifications were predicted in silico analysis by using Expasy tools (http://www.expasy.org/tools/), CrCDPK3 is probably a soluble protein present in the flagellar matrix.

Immunostaining of *Chlamydomonas* expressing CrCDPK3-HA with anti-HA antibody showed CrCDPK3 is scattered in the cell body and along the flagella (Figure 2E). In control cells, immuonstaining with anti-HA antibody did not show staining in the flagella though basal staining in the cell body was detected.

### Phototaxis, flagellar motility and mating is normal in RNAi strains of CrCDPK3

**Figure 3:**
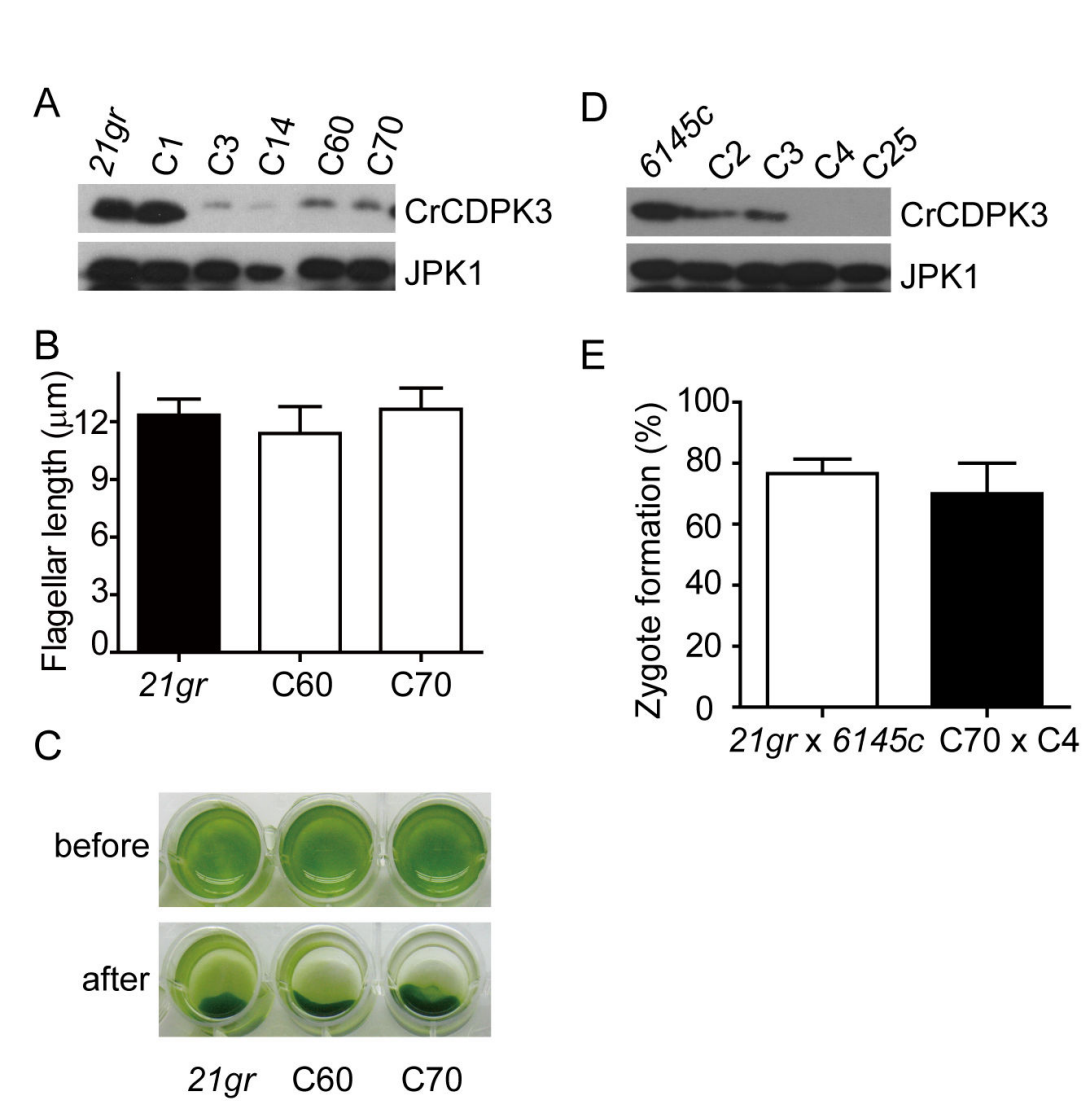
Phenotypic analysis of flagellar length, phototaxis and mating in CrCDPK3 RNAi strains. (A) Examination of CrCDPK3 protein level in RNAi strains. CrCDPK3 expression was analyzed by immunoblotting with anti-CrCDPK3 and anti-JPK1 antibodies. JPK1 was used as loading control. (B) Flagellar length measurements of wild type and two RNAi strains. Data are expressed as means ± SD in this and following figures. (C) Assay of phototaxis in 24-well microtiter plates. Note, cells accumulate on one side of the well after illumination. (D) Wild type 6145C (mt^-^) strains were transformed with miRNA constructs to generate RNAi strains in mt^-^ background. Protein expression of CrCDPK3 was examined by immunoblotting with JPK1 as loading control. (E) Rate of zygote formation. Zygote formation was scored 30 min after mixing mt^+^ and mt^-^ gametes generated from either pairs of wild types or RNAi strains.

To further study the function of CrCDPK3, *CrCDPK3* expression was knocked down by using artificial microRNA approach [34]. Immunoblot analysis of *Chlamydomonas* transformants with artificial microRNA construct had identified several strains with reduced expression of CrCDPK3 (Figure 3A). The RNAi strains possessed flagella of normal length (Figure 3B) and cells swam normally as wild type cells (data not shown). *Chlamydomonas* undergoes phototaxis which is thought to be regulated by differential sensitivities of the two flagella to intracellular calcium [8]. In response to calcium changes, differential activation of the two flagella causes swimming cells to turn [9]. To determine possible involvement of CrCDPK3 in phototaxis, wild type cells and RNAi strains in 24 well plates were illuminated from one side. All the samples showed similar phototaxis (Figure 3C), indicating CrCDPK3 may not be functioning in calcium-regulated phototaxis.

We next examined whether CrCDPK3 was involved in calcium-dependent mating that requires flagellar adhesion. Flagellar adhesion of gametes of opposite mating types triggers a signaling cascade leading to increase of cAMP level followed by cell-cell fusion to form zygotes [35], [36]. Alteration of calcium homeostasis by a variety of inhibitors inhibits cAMP rise and mating [12], [13] and activation of adenylate cyclase requires calcium [37]. In addition, this activation also requires modulation of protein phosphorylation activities [38], [39]. To test the role of CrCDPK3 in mating, RNAi strains in mating type minus background were generated by transformation of wild type strain 6145C mt^-^ with CrCDPK3 RNAi construct (Figure 3D). RNAi strains C70 mt^+^ and C4 mt^-^ were induced to form gametes in nitrogen free medium under continuous light. When gametes from these stains were mixed, cells agglutinated normally as wild type (data not shown). Furthermore, the percentage of zygotes formed was similar to that of wild type (Figure 3E). Thus, it appears that CrCDPK3 is not involved in calcium-dependent pathways during mating.

### CrCDPK3 forms an unknown complex in the flagella which is disrupted upon inducing flagellar shortening

**Figure 4:**
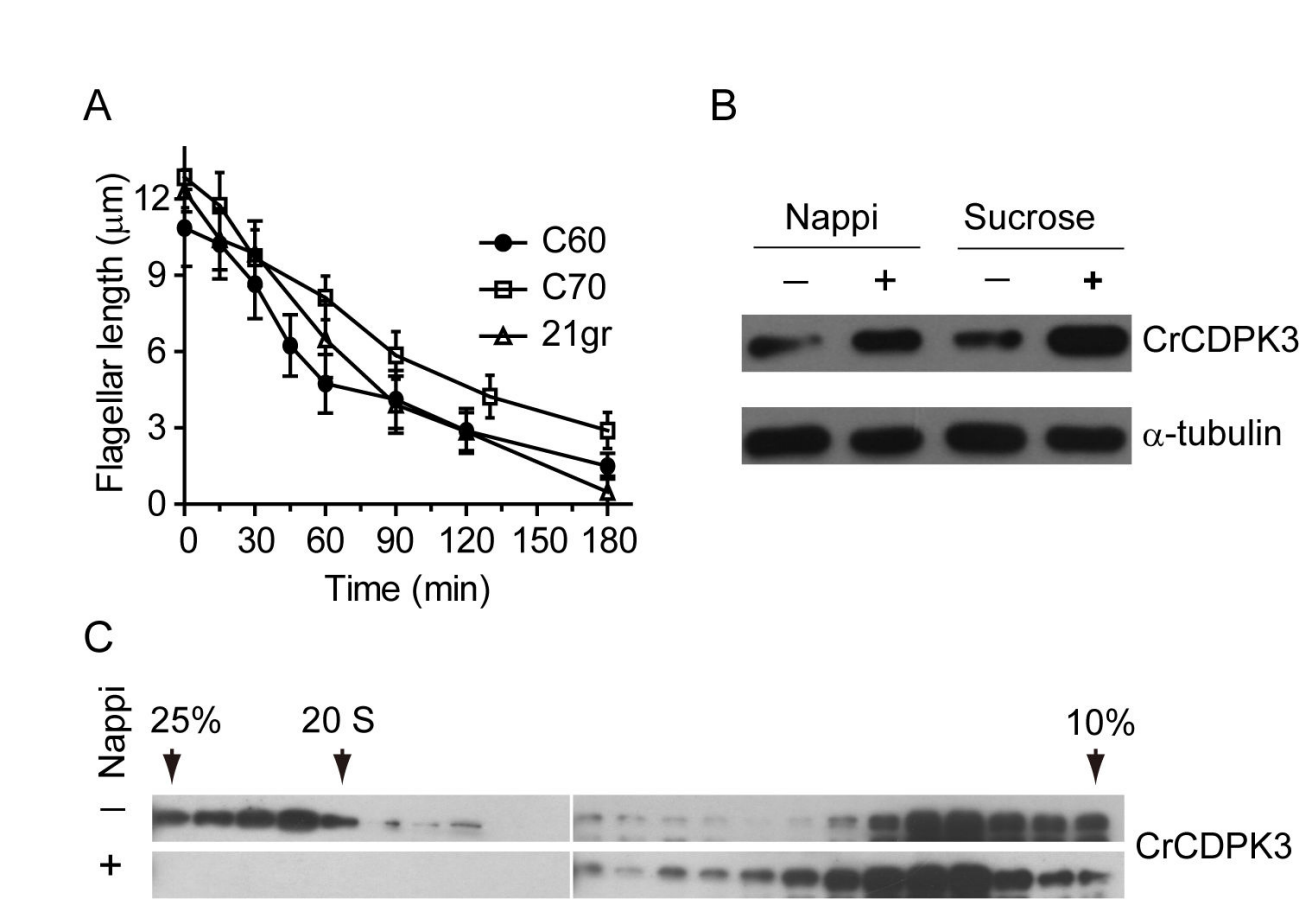
Characterization of CrCDPK3 during flagellar shortening. (A) Cells were treated with 20 mM NaPPi to induce flagellar shortening followed by cell fixation and flagellar length measurement. No apparent difference in shortening was observed between two RNAi and wild type strains. (B) Flagellar increase of CrCDPK3 upon induction of flagellar shortening. Cells were treated with 20 mM NaPPi or 0.2 M sucrose followed by flagellar isolation and immunoblotting with antibodies indicated. (C) Formation of flagellar CrCDPK3 complex and its disruption upon induction of flagellar shortening. Flagella were isolated from steady state cells and cells treated with 20 mM NaPPi for 10 min followed by extraction of membrane/matrix fractions, which were analyzed by a 10-25% sucrose gradient and immunoblotting. Note that CrCDPK3 formed a complex around 20 S in steady state flagella and was disrupted upon inducing flagellar shortening.

Various conditions including chelation of extracellular calcium or osmotic stress induce flagellar shortening, and reversal of flagellar shortening occurs by addition of excess calcium [14], [16] [40],. Flagellar shortening is regulated by protein kinase CALK [41], whose mammalian homologue Aurora A regulates cilia shortening and is activated by calcium [42]. In addition, a variety of protein kinases including MAP kinase and NIMA protein kinase regulates shortening of cilia and flagella [43]. To determine whether CrCDPK3 was involved in flagellar shortening, wild type and RNAi strains were treated with 20 mM sodium pyrophosphate (NaPPi) to induce flagellar shortening [14]. By measuring flagellar length at different times after treatment, we showed that flagellar shortening underwent similar kinetics in wild type and RNAi strains (Figure 4A). Similar results were obtained by inducing flagellar shortening with 0.2 M sucrose or 125 mM KCl (data not shown).

In spite of this, we analyzed property changes of flagellar CrCDPK3 during flagellar shortening. In steady state cells flagellar length is maintained by a balance between assembly and disassembly activities [44]. Activation of flagellar shortening pathway involves not only changes in signaling activity but also trafficking of a subset of proteins into the flagella. Intraflagellar transport proteins and CrKinesin13, a microtubule depolymerase that is required for flagellar shortening [45], increase several folds in the flagella upon triggering flagellar shortening [45], [46]. Flagella were isolated from steady cells and cells undergoing flagellar shortening induced by NaPPi or sucrose for 10 min from wild type cells and analyzed by immuoblotting. As shown in Figure 4B, flagellar increase of CrCDPK3 was observed upon flagellar shortening induced by both NaPPi and sucrose. To learn more about the property of CrCDPK3 during flagellar shortening, flagellar membrane/matrix fractions were subjected to sucrose gradient analysis followed by immunoblotting. Interestingly, CrCDPK3 formed an unknown protein complex around 20 S in flagella of steady state cells and this complex was disrupted upon flagellar shortening was induced (Figure 4C). The formation of protein complex in the flagella of steady state cells and its disruption upon inducing flagellar shortening indicates that this protein complex may function in flagellar assembly or preventing flagellar disassembly to maintain flagellar length.

### CrCDPK3 participates in flagellar assembly

**Figure 5:**
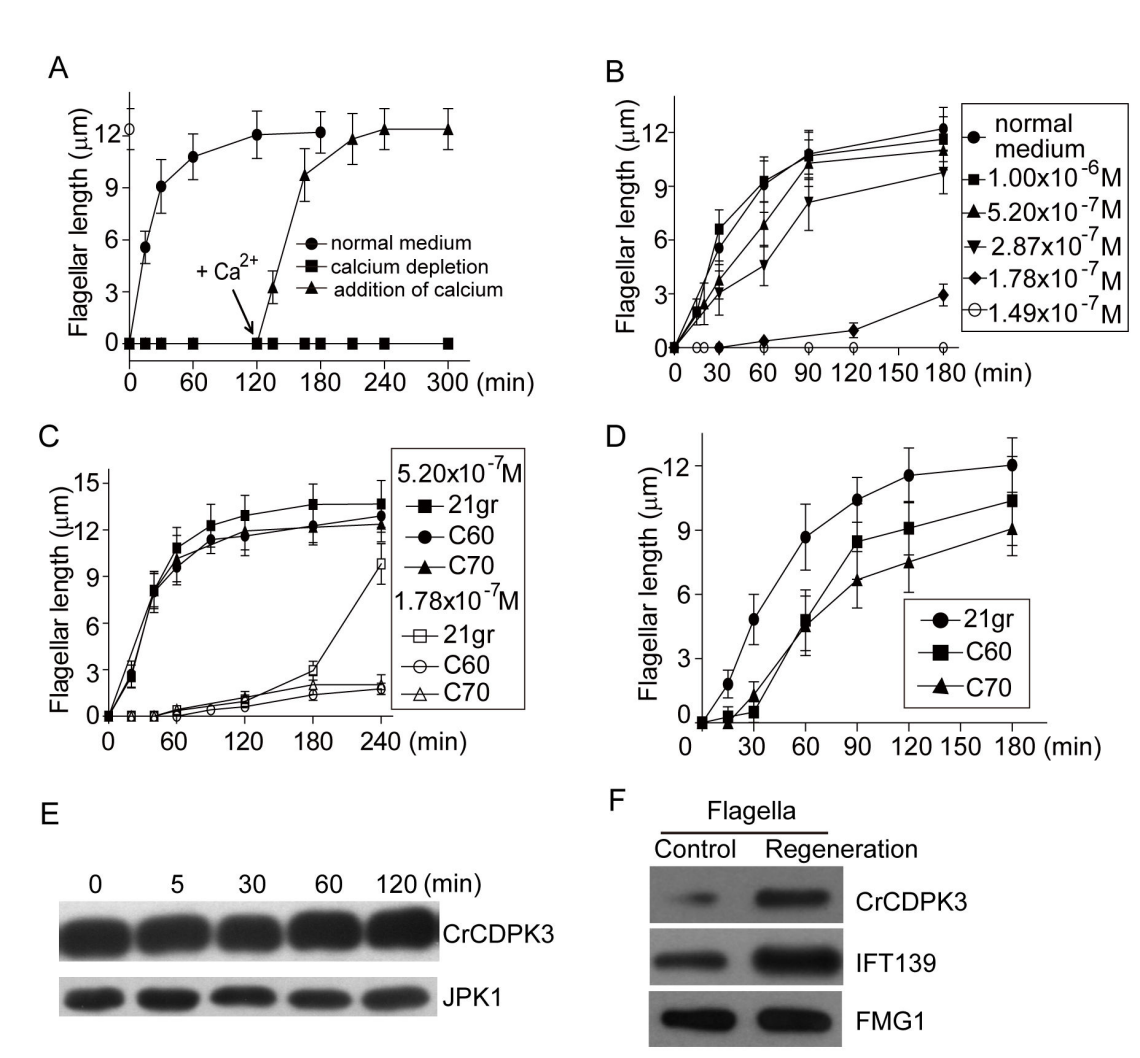
Requirement of calcium and CrCDPK3 for flagellar regeneration. (A) After deflagellation by mechanical shearing, 2.3 mM EGTA (final concentration) was added to the cell samples or not. At 120 min after deflagellation, CaCl_2_ was added to EGTA treated samples to reach 0.36 mM calcium present in normal medium. Samples were fixed at different times for flagellar length measurement. (B) Titration of [Ca^2+^]_e_ to determine calcium-dependent flagellar regeneration. After deflagellation, flagellar regeneration was allowed to proceed at different [Ca^2+^]_e_s. (C) Flagellar regeneration of CrCDPK3 RNAi strains at lower [Ca^2+^]_e_s. (D) Flagellar regeneration after transferring cells grown on agar plates into liquid medium. (E) Cell samples before (time 0) and at different times during flagellar regeneration after deflagellation were subjected to immunoblot analysis with anti-CrCDPK3 and anti-JPK1 antibodies. (F) Immunoblot analysis of CrCDPK3 in flagella from steady state cells and cells undergoing flagellar regeneration for 20 min. Equal flagelar proteins were loaded. IFT139 was used as positive control, which was shown to increase in regenerating flagella, and FMG1 used as loading control.

Next we examined any possible role of CrCDPK3 in flagellar assembly during flagellar regeneration. After deflagellation, cells rapidly regenerate flagella within 2 hrs [47]. When extracellular calcium is lowered to below 10^-6^ M, flagellar regeneration is delayed or prevented and occurs when calcium is restored [15], [16] [48],. To confirm this result, extracellular calcium level ([Ca^2+^]_e_) was lowered to below 10^-8^ M after deflagellation by mechanical shearing. Flagellar regeneration occurred normally in regular medium which contains 0.36 mM calcium (Figure 5A). As expected, flagellar regeneration was blocked in calcium depleted medium and occurred after addition of calcium. To examine [Ca^2+^]_e_ required for flagellar regeneration, 2.3 mM EGTA was added immediately after deflagellation followed by adding different amounts of CaCl_2_ to achieve different [Ca^2+^]_e_s and flagellar regeneration was then monitored at different times after deflagelation. At [Ca^2+^]_e_ higher than 1 x 10^-6^ M, flagellar regeneration was apparently normal (Figure 5B), which is consistent with previous report [15]. Within the 10^-7^ M range of [Ca^2+^]_e_, flagellar regeneration was affected to different extent. When the [Ca^2+^]_e_ was lowered to below 1.78 x 10^-7^ M, flagellar outgrowth was completely blocked (Figure 5B). This demonstrates that flagellar regeneration is sensitive to a small window of [Ca^2+^]_e_. To determine whether CrCDPK3 was involved in flagellar regeneration, control cells and RNAi cells were deflagellated and allowed to regenerate flagella at different [Ca^2+^]_e_s. The flagellar regeneration kinetics of RNAi cells was similar to that of wild type cells at [Ca^2+^]_e_ of 5.2 x 10^-7^ M (Figure 5C). At [Ca^2+^]_e_ of 1.78 x 10^-7^ M, wild type cells eventually regenerated almost full length flagella though exhibiting delayed flagellar regeneration. In contrast, two RNAi strains at this concentration of calcium showed severe defects in flagellar growth. This result indicates that CrCDPK3 only affects flagellar assembly at a small [Ca^2+^]_e_ window.

*Chlamydomonas* cells grown on agar plates do not grow flagella and regenerate flagella when transferred to liquid medium [41], [49]. The underlying mechanism is unknown. One speculation is that the micro-environment surrounding cell mass after growth might have depleted calcium in the medium, and upon transferring to liquid medium, cellular calcium homeostasis is changed that allows flagellar regeneration. We tested flagellar regeneration of CrCDPK3 RNAi strains upon transferring to liquid medium. Compared to wild type cells, RNAi strains showed delay and decreased rate of flagellar regeneration (Figure 5D).

The involvement of CrCDPK3 in flagellar assembly may be reflected in its property changes during flagellar regeneration. Cell samples after deflagllation and during flagellar regeneration were examined by immunoblotting with CrCDPK3 antibody. We failed to observe changes of protein amount, nor molecular weight shift of CrCDPK3, which often implicates protein phosphorylation (Figure 5E). However, we did observe flagellar enrichment of CrCDPK3. Flagella isolated from steady state cells and cells undergoing flagellar regeneration were subjected to immunoblot analysis. FMG1 was used as loading control. As expected, intraflagellar transport (IFT) proteins represented by IFT139 were increased in regenerating flagella (Figure 5F) [50]. Similarly, CrCDPK3 was also increased. Thus, CrCDPK3 requirement for flagellar regeneration and the associated property changes provide a link for calcium-regulated flagellar assembly.

### CrCDPK3 is defective in flagellar elongation induced by LiCl

**Figure 6:**
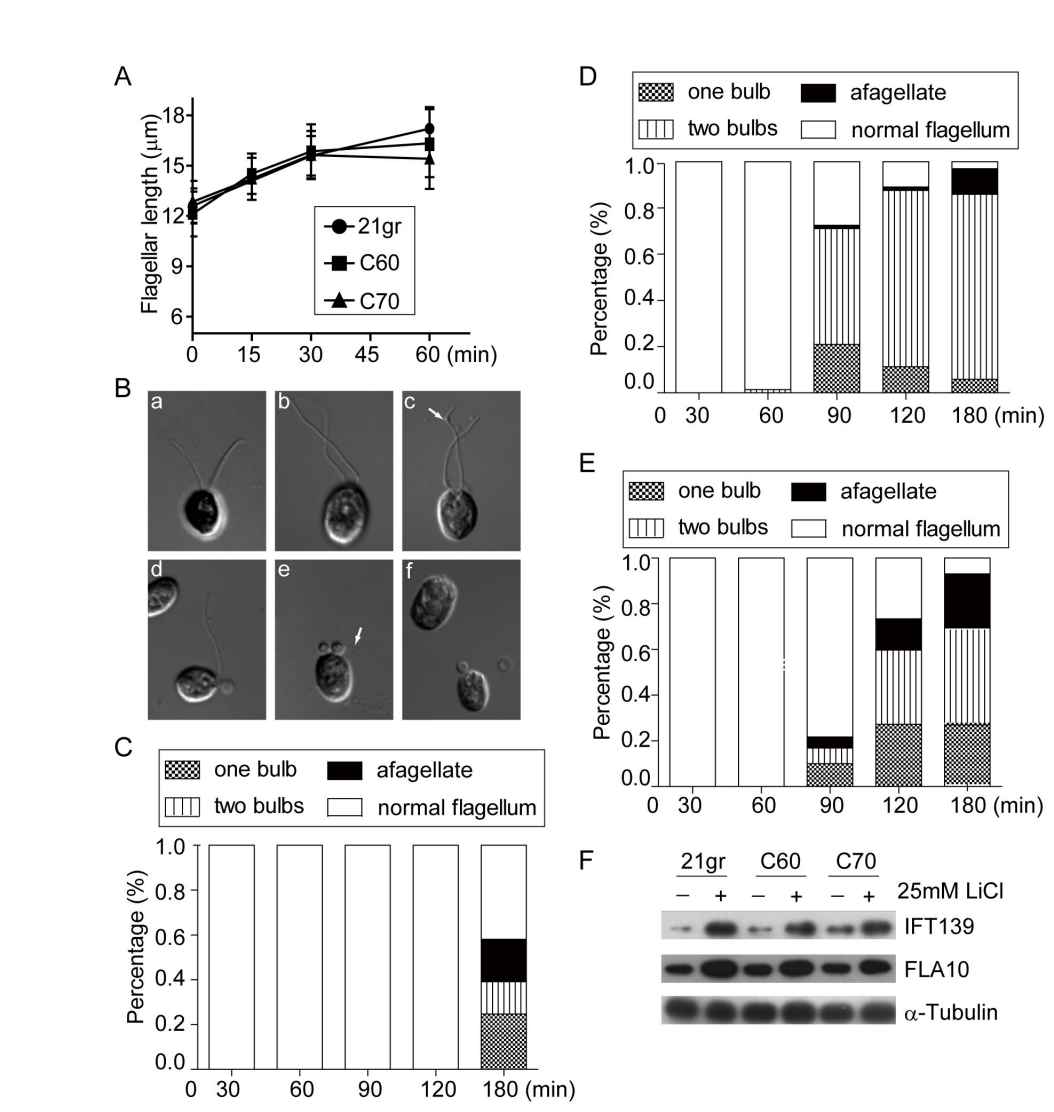
CrCDPK3 is defective in flagellar elongation induced by LiCl. (A) Flagellar length increase after treatment with 25 mM LiCl for wild type and RNAi strains. (B) Differential interference contrast images of cells before treatment (a) and 180 min after treatment (b–f). b, elongated normal flagella; c, flagellum with one small bulb proximal to flagellar tip (arrow); d, one flagellum curled at the flagellar base; e, two curled flagella with one remaining flagellar tip (arrow); f, loss of curled flagella. (C) Statistical presentation of cells with different forms of flagella after LiCl treatment for wild type cells, and (D) for RNAi strain C60, and (E) for RNAi strain C70. (F) Increase of IFT proteins during flagellar elongation induced by LiCl. Flagella isolated from wild type or RNAi strains after 30 min treatment were analyzed by immunoblotting with antibodies against IFT139, IFT motor protein Fla10, and α-tubulin, which was used as loading control.

To further confirm possible role of CrCDPK3 in flagellar assembly, we examined flagellar elongation induced by LiCl in CrCDPK3 RNAi strains. LiCl has been shown to stimulate cilia elongation in vertebrate cells [51] and *Chlamydomonas* [52], [53] [54],. Inhibition of GSK3β [52] or adenylate cyclase [51] has been proposed to underlie the effect of LiCl. Treatment of *Chlamydomonas* wild type cells with 25 mM LiCl induced about 40% increase of flagellar length from 12 µm to 17 µm similarly as reported (Figure 6A) [53]. Similar treatment of RNAi strains did not show apparent length difference. It has been reported that longer treatment of cells induces formation of bulbed flagella at the flagellar tip, and the flagella begin to curl at the base forming big “bulbs” which are eventually lost forming aflagellate cells [52], [53]. We have confirmed this observation, as shown in Figure 6B and C. By careful examination, it appears that flagellar bulb initially formed at a region distal to the flagellar tip (Figure 6B, panel c), which might lead to flagellar curling and form curled flagella at the base (Figure 6B, panels d, e, f). In complete curled flagella, apparent normal flagellar tip could also be observed (Figure 6B, panel e, arrow). By examination of RNAi strains, we found that there was an early onset of “bulb” formation in both RNAi strains (Figure 6D and E). Though the percentage of “bulbs” formation in two RNAi strains were slightly different, as early as 90 min after treatment, cells started to form “bulbs” in both strains. And at 120 min when wild type cells showed normal flagellar morphology, around 80% of cells in RNAi strains were defective.

The mechanism of flagellar “bulb” formation is not known. The curled flagella are likely formed by curling the axoneme within the flagellar membrane. Flagellar growth requires IFT to deliver flagellar precursors to the tip for their incorporation into the axoneme [55], [56] [57], [58], and flagellar membrane biogenesis has to be accordingly coordinated [59], [60]. Defects in either or both pathway(s) may result in changes of flagellar morphology. LiCl has been shown to increase IFT proteins in the flagella [52]. To examine whether CrCDPK3 affected IFT, isolated flagella from wild type and two RNAi strains after LiCl treatment were examined for IFT proteins by immunoblotting. Data in Figure 6F shows that flagellar IFT increase in the RNAi strains were apparently the same as that of wild type cells. It remains to be determined whether other aspects of IFT such as cargo loading or IFT turnaround at the tip are affected.

## Discussion

This is the first attempt to examine physiological functions of CDPKs in regulating flagellar related activities. In this work, we have identified 14 CDPKs in the *Chlamydomonas* genome and studied the function of CrCDPK3, a flagellar localized CDPK. The presence of large numbers of CDPKs in *Chlamydomonas* may not be surprising. 34 CDPKs are identified in *Arabidopsis* and most of the known calcium-stimulated kinase activities are associated with CDPKs [31] Several explanations may account for this. First, higher plants as well as algae cannot escape from fluctuating environments. It has been shown that alteration of various environmental conditions including cold, osmotic stress, and salt stress induces changes of calcium homeostasis [61]. The calcium changes are specific to a given stress in terms of cellular localization, magnitude and duration, different calcium waveforms or spikes [61], [62]. All these calcium signatures have to be decoded by different calcium sensors and effectors to accordingly regulate cell growth, cell morphology, metabolism and gene expression in response to stress or initiate new developmental programs [24]. Second, the major classes of calcium effectors CaMK [20] and PKC [21] present in animals are rare [22] or not present in plants [23]. In *Chlamydomonas* , CaMK is implicated in flagellar beating, yet the genes have not been identified [63], and no molecular evidence for PKC is present to our knowledge.

The function of CDPKs has been extensively studied in higher plants and apicomplexans. In higher plants, CDPKs have been implicated in abiotic stress, development and hormone response (reviewed in 22). In apicomplexan parasites, cell motility, developmental transitions are regulated by CDPKs (reviewed in 26). Though apicomplexans have flagella or cilia and CDPKs activities have been detected in *Paramecium* cilia [27], [28], no flagellar or ciliary function of CDPKs has been studied. This work expands new functions of CDPK protein kinase family.

In the *Chlamydomonas* flagellar proteome, three CDPKs including CrCDPK1, 3 and 11 are identified [30]. We showed that CrCDPK3 is indeed a flagellar protein evidenced by immuoblotting of isolated flagella and immuostaining. Interestingly, it is localized predominantly in the flagelar membrane/matrix, which is consistent with the presence of CDPK activity in the membrane/matrix demonstrated by in vitro phosphorylation assay [19].

We have used artificial miRNA approach to knock down CrCDPK3 expression to examine its flagellar related functions since gene knockout techniques have not been developed in this organism [34]. CrCDPK3 RNAi strains reduced CrCDPK3 level around 80%. No apparent effects were observed on phototaxis as well as flagellar motility. Flagellar motility has been shown to be regulated by CaMK [63]. We also examined possible role of CrCDPK3 in mating. It has been proposed that intraflagellar increase of calcium induced by flagellar adhesion regulates activation of adenylate cyclase to generate cAMP [12], which triggers all the mating response including cell wall loss, flagellar tip activation, and protrusion of mating organelles [35], [36]. We failed to observe obvious effects of CrCDPK3 on flagellar adhesion as well as rate of zygote formation. It has been reported that protein phosphorylation activities are upstream of activation of adenylate cyclase [39], it remains to be demonstrated whether calcium-dependent phosphorylation is required. Though the effects of CrCDPK3 on flagellar motility and mating are both negative, we could not discount the possibility that CrCDPKs including CrCDPK3 are involved because flagellar CDPKs may play redundant roles and the residual amount of CrCDPK3 in the RNAi strains may be sufficient for functioning in these processes.

Our data indicate that CrCDPK3 is likely involved in flagellar assembly. First, CrCDPK3 increases in the flagella during flagellar regeneration. Second, upon inducing flagellar shortening, pre-formed complex of CrCDPK3 is disrupted. Third, flagellar elongation induced by LiCl in the CrCDPK3 RNAi strains is compromised compared to wild type cells. Lastly, at low calcium level flagellar regeneration is prevented when CrCDPK3 expression is knocked down. After flagellar loss, cells rapidly regenerate flagella within 2 hrs [47]. Lowering extracellular calcium level to below 10^-6^ M delays or prevents flagellar regeneration [15], [16], which is consistent with our data. Interestingly, knockdown expression of CrCDPK3 affects flagellar regeneration only at a small window of lower calcium level. One likely explanation is that at elevated calcium level, increased activity of residual CrCDPK3 present in the RNAi cells may compensate for the loss of CrCDPK3. It has been shown that CDPKs exhibit different sensitivities to calcium level [24]. Another possibility is that other flagellar CDPKs regulate flagellar assembly at higher calcium level.

Flagellar assembly requires coordination of several cellular processes including gene expression (reviewed in 64), mobilization of cytoplasmic flagellar precursors [64], [65], delivery of flagellar precursors by IFT to the assembly sites [55], [56] [66], and incorporation of flagellar precursors at the flagellar tip [57], [58]. These processes must be coordinated by signaling events since flagellar length induces protein phosphorylation changes [67] and alteration of expression of protein kinase genes affect flagellar length [43], [68]. CrCDPK3 might be involved in any of these processes. At lower calcium level, gene expression associated with flagellar regeneration and flagellar regeneration itself are both blocked [15]. Since in the absence of protein synthesis, flagella are still able to assemble approximately half length with regular kinetics [47], the role of calcium cannot be solely attributed to gene expression. IFT trafficking is unlikely affected by CrCDPK3 since IFT increase appears normal in CrCDPK3 RNAi cells during flagellar elongation induced by LiCl. Interestingly, CrCDPK3 forms an unknown complex in the flagellar membrane/matrix with a similar size to radial spoke precursors [66]. Thus, one possible function of CrCDPK3 is in regulating cargo loading of radial spoke precursors into IFT complexes or their incorporation into flagellar axonemes.

## Materials and Methods

### Strains, cell culture and special chemicals

*Chlamydomonas reinhardtii* strains *21gr* (mt+) (CC-1690), *6145c* (mt-) (CC-1691), are available from the *Chlamydomonas* Genetics Center, University of Minnesota. Growing of vegetative cells, gametogenesis and mating are described previously [45]. Briefly, vegetative cells were grown in M-medium in 250 ml Erlenmeyer flasks with air bubbling in 14: 10 h light-dark cycle at 23 ^o^C. Gametogenesis was induced in nitrogen-free *M* *medium* for 12-18 hrs in continuous light. Sodium pyrophosphate (NaPPi) and LiCl (Sigma, St. Louis, MO) were used at 20 mM and 25 mM, respectively.

### Analysis of flagella-related phenotypes

To analyze phototaxis, cell cultures placed in 24 well plates were illuminated on one side followed by examination of cell accumulation on the other side [32]. Images were taken by using regular digital camera. Flagellar motility was manually examined under Zeiss Axio Observer Z1 microscope (Carl Zeiss, Inc., Germany) with a 40 x objective lens. For mating, equal numbers of gametes of opposite mating types were mixed together and allowed to form zygotes. Flagellar adhesion and zygote formation were scored microscopically [69].

Deflagellation, flagellar regeneration, induction of flagellar shortening by NaPPi, and flagellar length measurement are essentially as described [45], [67]. Deflagellation was induced by mechanical shearing unless noted otherwise [45], [70]. An Ultra-Turrax homogenizer (model IKA T10 basic, IKA, Guangzhou, China) was set at scale 4 and 25 ml cells with cell density of 1 x 10^7^ cells/ml were processed in a 50 ml conical tube. Flagellar regeneration was allowed to proceed at different calcium concentrations. [Ca^2+^]_e_s were adjusted by adding 2.3 mM EGTA (final concentration), pH 7.5, to the medium followed by adding different amounts of CaCl_2_ and the [Ca^2+^]_e_s were estimated by using published method [71], which was used previously in *Chlamydomonas* [15]. Specifically, CaCl_2_ with final concentrations of 3, 1.5, 1.25, 1.0 and 0.9 mM were added to the medium after adding 2.3 mM EGTA to make [Ca^2+^]_e_ of 1.00 x 10^-6^ M, 5.20 x 10^-7^ M, 2.87 x1 0^-7^ M, 1.78 x 10^-7^ M, and 1.49 x 10^-7^ M. For flagellar regeneration from cells grown on agar plates, cells were first grown on 1.5% agar plate for 5 days. Cells from plates were scratched into small amount of cold medium and separated into individual cells. Flagellar regeneration was allowed by adding medium of room temperature. To induce flagellar shortening, 20 mM NaPPi or 0.2 M sucrose was used [14], [40]. Flagellar elongation was induced by adding 25 mM LiCl [52]. For flagellar length measurement, cells were fixed in 1% glutaraldehyde at different times after treatments and imaged on a Zeiss microscope as described above equipped with a QuantEM 512SC camera (Photometrics, Huntington Beach, CA). Flagellar length was measured by using ImageJ (NIH, Bethesda, MD) and calibrated with a micrometer. For each measurement, flagella from at least 50 cells were scored.

### Flagellar isolation, fractionation and sucrose analysis

Flagella were isolated after deflagellation by pH shock as previously described [46]. The flagllar pellet was dissolved in buffer A (20 mM HEPES, pH 7.2, 5 mM MgCl_2_, 1 mM DTT, 1 mM EDTA) containing EDTA-free protease inhibitor cocktail (Roche), 25 µg/ml ALLN and 0.5% NP40 and stored in liquid nitrogen. For fractionation of membrane/matrix and axonemal fractions, flagella were thawed on ice and centrifuged at 14000 rpm for 10 min at 4^o^C in a table top centrifuge (Model 5417R, Eppendorf). The supernatant was taken as membrane/matrix fraction and the pellet after wash once with buffer A by short spin as axonemal fraction [72]. 10-25 percentage points sucrose gradient was used for analysis of flagellar membrane/matrix proteins and fractionated into 23-24 fractions [46]. The sedimentation value of the CrCDPK3 complex was estimated based on the fractions from similar sucrose gradient analysis [66], [46].

### Nucleotide acid manipulation, bacterial protein expression and *Chlamydomonas* transformations

HA-tagged CrCDPK3 construct for expression in *Chlamydomonas* , GST and GST-CrCRCDPK3 constructs for expression in bacteria and miRNA construct were made by using general molecular techniques. All the constructs were verified by sequencing. A full-length cDNA of CrCDPK3 was cloned by PCR and cloned into pMD19-T vector (TAKARA). To make HA-tagged construct for ectopic gene expression of CrCDPK3 in *Chlamydomonas* , a 1.3 kb genomic DNA fragment upstream of start codon was used as promoter and fused with CrCDPK3 cDNA. The 3xHA tag was inserted into the 3’ end before stop codon followed by rubisco terminator. For antibiotic selection of *Chlamydomonas* transformants, expression cassette of paromomycin resistant gene *aphVIII* from plasmid pIS103 [73] was cloned into the above plasmid. This final construct was linearized with DraI before transformation.

For bacterial expression of GST-CrCDPK3, full-length cDNA was inserted into SmaI and HindIII sites of GST expression vector pPGH, a derivative of pPGX-4T-2. pPGH was used to express GST. Both pPGH and pPGH-CrCDPK3 plasmids were transformed into *E. coli* BL21 cells for protein expression. Expressed proteins were purified by using Glutathione–Agarose beads following instructions (Sigma).

*Chlamydomonas* cells were transformed by using electroporation [74]. Cells were grown in TAP medium with air bubbling at 23 ^o^C under continuous light for 3-4 days until cell density reached around 5 x 10^6^ cells/ml. The cells were then inoculated into a 250 ml Erlenmeyer flask containing 150 ml TAP medium at cell density around 1 x10^6^ cells/ml and cultured for one day with shaking (200 rpm) at 23 ^o^C under continuous light. The cells were finally resuspended in TAP medium containing 60 mM sorbitol to a final concentration of 1x10^8^ cells/ml. 250 µl cells containing 100 ng DNA were electroporated in an electroporator (Model ECM 630, BTX). The electroporation parameters used were 800V voltage, 1575Ω resistance and 50 µF capacitance. After electroporation, cells were immediately cooled down on ice for 10 min and transferred into a 50 ml tube containing 10ml TAP medium. After shaking overnight in the dark for recovery, the cells were collected by centrifugation, resuspended in 3 ml 20% corn starch in TAP medium and then plated onto 1.5% agar selection plates containing 10 µg/ml paramomycin.

### Gene silencing by using artificial miRNA

Artificial miRNA construct was designed by using the procedures from WMD3 – Web MicroRNA Designer (http://wmd3.weigelworld.org). The target sequence is GCGTCAATACTCGAAGTTCTT in the 3’ coding region. Multiple rounds of overlapping PCR were carried out to generate a DNA fragment to replace the SpeI and XbaI fragment of the pChlamiRNA3int vector [34]. The primers used were: amiRNA-SpeI-F (GACTAGTGCGTCAATACTCGAAGATCTATCTCGCTGATCGGCACCATGGGGGTGGTGGT); amiRNA-SpeI-R (TACTAGCGCGTCAATACTCGAAGTTCTATAGCGCTGATCACCACCACCCCCATGGTGCC); amiRNA-XbaI-F (CGAGTATTGACGCGCTAGTAGCCGGAACACTGC); and amiRNA-XbaI-R (CTGCTGCCATCTAGAGGTG). The pChlamiRNA3int-CDPK3 construct was linearized with DraI and transformed by electroporation into *Chlamydomonas* . The transformants were screened by immunoblotting with antibodies against CDPK3 and JPK1. JPK1 was used as control.

### SDS-PAGE and immunoblotting

The procedures were essentially as described [45]. *Chlamydomonas* cell or flagellar samples were dissolved in buffer A containing EDTA-free protease inhibitor cocktail (Roche) and 25 µg/ml ALLN and boiled in 1 x SDS sample buffer for 5 min before being subjected to SDS-PAGE analysis. Primary antibodies used are as follows: rat anti-HA (1:3,000) (clone 3F10, Roche), mouse anti-α-tubulin (1:10,000) (DM1A, sigma), mouse anti-FMG1 (1:5,000) (kindly provided by Dr. Bloodgood), rabbit anti-JPK1 (1:3,000), rabbit anti-CrCDPK3 (1:5,000), mouse anti-IFT139 (1:5,000) and rabbit anti-FLA10 (1:1000) (kindly provided by Dr. Cole). Rabbit anti-CrCDPK3 antibody was made against bacterial expressed His-tagged CrCDPK3 (1-202 amino acids) by Abmart, China.

### Immunofluorescence Microscopy

Immuostaining method was essentially as described previously [65]. Primary antibodies used were anti-α-tubulin (1:200) and anti-HA (1:100), and secondary antibodies Texas Red goat anti-mouse IgG (1:200) and Alexa Fluor 488 goat anti-rat IgG (1:200) (Molecular Probes). Samples were imaged on a Zeiss780 Observer Z1 Confocal Laser Microscope (Zeiss, Germany). Images were acquired and processed by ZEN 2011 Light Edition software and Photoshop, and assembled in Adobe Illustrator (Adobe, USA).
